# Integrative analysis of circulating tumor cells (CTCs) and exosomes from small‐cell lung cancer (SCLC) patients: a comprehensive approach

**DOI:** 10.1002/1878-0261.13765

**Published:** 2024-11-22

**Authors:** Dimitrios Papakonstantinou, Argyro Roumeliotou, Evangelia Pantazaka, Athanasios‐Nasir Shaukat, Athina Christopoulou, Angelos Koutras, Foteinos‐Ioannis Dimitrakopoulos, Vassilis Georgoulias, Anastasia Xagara, Evangelia Chantzara, Fillipos Koinis, Athanasios Kotsakis, Constantinos Stathopoulos, Galatea Kallergi

**Affiliations:** ^1^ Laboratory of Biochemistry/Metastatic Signaling, Section of Genetics, Cell Biology and Development, Department of Biology University of Patras Greece; ^2^ Department of Biochemistry, School of Medicine University of Patras Greece; ^3^ Oncology Unit ST Andrews General Hospital of Patras Greece; ^4^ Clinical and Molecular Oncology Laboratory, Division of Oncology, Department of Medicine, Medical School University of Patras Greece; ^5^ Hellenic Oncology Research Group (HORG) Athens Greece; ^6^ Department of Medical Oncology General University Hospital of Larissa Greece

**Keywords:** circulating tumor cells, exosomes, liquid biopsy, lung cancer, miRNAs

## Abstract

The increased metastatic ability of small‐cell lung cancer (SCLC) necessitates the identification of new prognostic biomarkers for clinical evaluation during the disease course. Our previous research highlighted the clinical relevance of transcription factor JunB (JUNB), C‐X‐C chemokine receptor type 4 (CXCR4), and programmed cell death 1 ligand 1 (PD‐L1) in breast and non‐small cell lung cancer (NSCLC) patients. In the current study, we examined these biomarkers in circulating tumor cells (CTCs) and plasma‐derived exosomes from 100 treatment‐naïve SCLC patients. CTCs were analyzed using the VyCAP system, whereas exosomes were characterized molecularly and transcriptomically. JUNB, CXCR4, and PD‐L1 were highly prevalent in CTCs. Patients exhibited significantly increased protein exosomal expression of JUNB and CXCR4 compared to healthy individuals. Overexpression of JUNB and CXCR4 in exosomes can distinguish patients from normal donors, offering an interesting tool for early diagnosis. The presence of JUNB and/or CXCR4 in CTCs correlated with significantly poorer overall survival. CXCR4 exosomal overexpression was associated with CTC presence and their phenotypes. Conclusively, a comprehensive analysis of CTCs and exosomes provides useful prognostic and potential diagnostic tools for SCLC patients.

## Graphical abstract

This study simultaneously investigated circulating tumor cells (CTCs) and exosomes from small‐cell lung cancer (SCLC) patients. The elevated expression of JUNB and CXCR4 in CTCs was a poor prognostic factor for SCLC patients, whereas exosomal overexpression of these biomarkers revealed a high discrimination ability of patients from healthy individuals, providing a potentially interesting tool for early diagnosis.

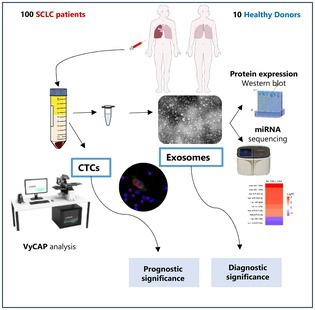

**Abbreviations**
Ab: antibody
ANOVA: analysis of variance
AP‐1: activator‐protein 1
CK: cytokeratin
CTCs: circulating tumor cells
ctDNA: circulating tumor DNA
DFS: disease‐free survival
DTCs: disseminated tumor cells
EMT: epithelial‐to‐mesenchymal transition
EP: exosomal protein
EVs: extracellular vesicles
exoMIRs: exosomal microRNAs
FBS: fetal bovine serum
GO: Gene Ontology
HD: healthy donor
HIF: hypoxia‐inducible factor
HS: high sensitivity
miRNAs: microRNAs
NSCLC: non‐small cell lung cancer
OS: overall survival
PBMCs: peripheral blood mononuclear cells
PD‐1: programmed cell death protein 1
PD‐L1: programmed death ligand 1
PFS: progression‐free survival
ROC: receiver operating characteristic
SCLC: small‐cell lung cancer
STR: short tandem repeat
TEM: transmission electron microscopy
TGF‐β: transforming growth factor‐beta
TME: tumor microenvironment
TNBC: triple‐negative breast cancer

## 1 Introduction

Lung cancer is the leading cause of cancer‐related mortality worldwide, affecting both men and women [1]. The main histological subtypes are small‐cell lung cancer (SCLC) and non‐small cell lung cancer (NSCLC) [2]. SCLC, a neuroendocrine carcinoma, accounts for about 15% of lung cancers and is noted for its aggressive progression and early metastasis [3].

SCLC is classified into limited disease, confined to regions near the primary tumor, and extensive disease, with metastasis to distant organs [4]. Approximately two‐thirds of patients are diagnosed with extensive disease, leading to a poor prognosis due to the lack of early detection tools [5]. As a result, the 5‐year overall survival rate is very low (around 5%), especially for patients with extensive disease [6]. This highlights the urgent need for new diagnostic tools to enable early detection [7].

Liquid biopsy has become an efficient, noninvasive method for assessing cancer biomarkers in various body fluids. It involves analyzing circulating tumor cells (CTCs), circulating tumor DNA (ctDNA), microRNAs (miRNAs), and extracellular vesicles (EVs) [8, 9, 10]. CTCs, major contributors to metastasis, can serve as real‐time biopsies. Their detection and enumeration are linked to poor outcomes in cancers such as breast, prostate, and lung [11, 12, 13]. CTCs from solid tumors can be identified in blood by epithelial markers like EpCAM and cytokeratins (CK) 8, 18, and 19. However, epithelial‐to‐mesenchymal transition (EMT) can render some CTC subgroups undetectable [14]. Hence, it is crucial to identify these subpopulations of CTCs with metastatic potential and evaluate their expression profile for significant biomarkers [15].

The programmed cell death protein 1/programmed death ligand 1 (PD‐1/PD‐L1) axis helps CTCs evade immune detection, crucial for metastasis. PD‐L1, which inhibits T‐cell activity, is expressed in various cells, including cancer cells [16]. Numerous studies have shown elevated PD‐L1 expression in CTCs from various cancers, highlighting its importance as a biomarker [17, 18, 19, 20, 21, 22, 23, 24, 25].

PD‐L1 expression is regulated by the JUNB transcription factor [26]. We previously identified JUNB and the chemokine receptor CXCR4 as prognostic biomarkers for breast [27, 28] and lung cancer [29]. JUNB, part of the AP‐1 family, regulates genes linked to invasion, angiogenesis, and metastasis [30, 31]. CXCR4, a G protein‐coupled receptor, is associated with tumor growth, metastasis, and survival [32, 33]. The presence of JUNB and/or CXCR4‐positive CTCs correlated with shorter overall survival (OS) and progression‐free survival (PFS) in metastatic breast and lung cancer patients [29]. Additionally, the (CK^+^CXCR4^+^JUNB^+^) phenotype in bone marrow disseminated tumor cells (DTCs) of early breast cancer patients is strongly linked to poorer OS [28].

EVs have garnered attention for their role in tumor progression and immune evasion [34, 35]. EVs, ranging in size from 30 to 150 nm, are released into various bodily fluids, such as blood, urine, and saliva, by a diverse array of cell types [36]. Exosomes, a subset of EVs, contain bioactive molecules such as proteins, lipids, and miRNAs that facilitate intercellular communication [37]. Tumor cells use EVs to transmit molecular contents to distant cells, aiding in the formation of the tumor microenvironment (TME) [38].

A growing emphasis is being placed key TME components [39, 40]. ExoMIRs can be transferred from cancer cells to other cells in the TME, influencing gene expression patterns, including those related to EMT and metastasis [41, 42, 43]. In this study, we examine JUNB, CXCR4, and PD‐L1 expression in CTCs of SCLC patients. Additionally, we present the first comprehensive analysis of simultaneous JUNB and CXCR4 expression in CTCs and plasma exosomes from SCLC patients.

## 2 Materials and methods

### 2.1 Cell culture and spiking experiments

The NCI‐H1299 (RRID: CCVL_0060) cell line was used for the evaluation of JUNB and CXCR4 expression and was obtained from the American Type Culture Collection (Manassas, VA, USA). H1299 cells were cultured in Dulbecco's modified Eagle medium with Glutamax (Thermo Fisher Scientific, Waltham, MA, USA) supplemented with 10% fetal bovine serum (FBS; PAN‐Biotech, Aidenbach, Germany) and 50 U·mL^−1^ penicillin/50 g·mL^−1^ streptomycin (Thermo Fisher Scientific). Cells were maintained at 37 °C in a humidified atmosphere of 5% CO_2_ in air, and subcultivation was performed with 0.25% trypsin–EDTA (Thermo Fisher Scientific). The cell line was authenticated using short tandem repeat (STR) profiling, and all experiments were performed with mycoplasma‐free cells.

For spiking experiments, as described in our previous work [29, 44], H1299 cells were spiked into healthy donors' (HD) peripheral blood mononuclear cells (PBMCs; 1000 H1299 cells/100000 PBMCs) and centrifuged at 448 *g* for 2 min on Superfrost glass slides (Thermo Fisher Scientific). Slides from spiking experiments were used as controls in order to evaluate the sensitivity and specificity of the method/antibodies.

### 2.2 Patients' CTC samples and cytospins' preparation

A total of 100 chemotherapy‐naïve SCLC patients were enrolled in the study. Patients' median age was 69 years (range 40–84), and 86% of them had extensive disease. The vast majority of patients were smokers or ex‐smokers. Patients received first‐line treatment with carboplatin plus etoposide and cisplatin plus etoposide, while some patients received durvalumab or atezolizumab (Table 1).

**Table 1:** Patients' clinicopathological characteristics. MR, minimal response; PD, progressive disease; PR, partial response; SCLC, small‐cell lung cancer; SD, stable disease.

Characteristic	All SCLC patients (*n* = 100)	
	No.	%
Age (years)		
Mean = 67.2		
Standard deviation = 8.1		
Median = 69		
Gender		
Male	75	75
Female	21	21
Unknown	4	4
Smoking status		
Active smokers	22	22
Ex‐smokers	30	30
Unknown	48	48
Disease stage		
Extended stage	77	77
Limited stage	13	13
Unknown	10	10
Tumor detection site		
Left lung	26	26
Right lung	31	31
Unknown	43	43
Best response to therapy		
PD	22	22
PR	14	14
SD	24	24
MR	0	0
Unknown	40	40
Survival status		
Alive	67	67
Dead	26	26
Unknown	7	7
First‐line treatment		
Carboplatin + etoposide	16	16
Carboplatin + etoposide + durvalumab	29	29
Carboplatin + etoposide + atezolizumab	7	7
Cisplatin + etoposide	2	2
Cisplatin + etoposide + durvalumab	2	2
Cisplatin + etoposide + atezolizumab	2	2
Unknown	42	42

From January 2021 to February 2023, peripheral blood from all patients, as well as from 10 HDs, was collected in EDTA K2 tubes. All blood samples were collected from the site of vein puncture, after discarding the first 5 mL, in order to avoid contamination with epithelial cells from the skin. This study adheres to the Declaration of Helsinki guidelines, as updated in 2013, and was approved by the Ethics and Scientific Committees of all involved institutions: Metropolitan General Hospital (35/00‐03/16); University General Hospital of Larissa (32 710/3‐8‐20); University General Hospital of Patras and Olympion Hospital of Patras (172/18‐09‐2020); and Saint Andrews General Hospital of Patras (521/13‐10‐2020). Patients and HDs gave their informed written consent for having their blood collected and for their clinical follow‐up data to be used for research purposes.

PBMCs from SCLC patients were isolated using Ficoll–Hypaque (*d* = 1.077 g·mol^−1^) density gradient centrifugation, as referenced in prior research of our group [29, 45]. Specifically, centrifugation was performed at 180 *g* for 30 min without brakes, with CTCs being contained within the fraction of PBMCs. Although this method is characterized by low CTC recovery rate [14], this was not a major limitation in our study, due to the high number of CTCs that patients with SCLC usually harbor in their blood, compared to other types of cancer [46]. Additionally, this method allowed the simultaneous isolation of plasma (utilized for exosomal isolation) and the CTCs. Additionally, Ficoll–Hypaque density centrifugation can be characterized as an EpCAM‐independent method as it enables the isolation of both epithelial and mesenchymal CTCs, detecting different states of EMT and isolating heterogeneous CTCs' subclones. After isolation, PBMCs were washed twice with PBS and centrifuged at 150 *g* for 10 min. Aliquots of 500 000 cells/500 μL were centrifuged at 450 *g* for 2 min on Superfrost glass slides (Thermo Fisher Scientific). Cytospins were dried up and stored at −80 °C until use. One slide from each patient was analyzed with the automated VyCAP system for the identification of CTCs and evaluation of the expression of JUNB and CXCR4, and one slide from each patient was analyzed for the evaluation of PD‐L1, with triple immunofluorescence stainings.

### 2.3 Triple immunofluorescence staining

Peripheral blood cytospin preparations from SCLC patients, and spiked samples, were analyzed using triple immunofluorescence, as mentioned in our previous studies [29, 44, 45]. Particularly, two triple immunofluorescence stainings were performed, using the corresponding antibodies (Ab) for CK, CXCR4, and JUNB and CK, PD‐L1, and CD45.

For the (CK/CXCR4/JUNB) staining, samples were incubated with PBS for 5 min followed by fixation with 3% paraformaldehyde for 30 min at RT and permeabilization with 0.5% Triton X‐100 for 10 min at RT. Nonspecific binding was avoided by blocking with 5% FBS in PBS at 4 °C overnight. Cells were then incubated with the mouse A45‐B/B3 Ab (Amgen, Southern Oaks, CA, USA), for the detection of CKs 8/18/19, for 1 h. Anti‐mouse Alexa 555 (Life Technologies, Carlsbad, CA, USA) was used as a secondary Ab for 45 min. Samples were further incubated with the rabbit anti‐CXCR4 Ab (ABCAM, Cambridge, MA, USA) for 1 h followed by staining with the secondary anti‐rabbit Alexa 647 Ab (Life Technologies) for 45 min. Cells were then incubated for 1 h with the mouse anti‐JUNB Ab conjugated with Alexa 488 (Santa Cruz Biotechnology, Santa Cruz, CA, USA). Finally, slides were mounted on Prolong antifade medium containing DAPI for nuclear visualization.

For the (CK/PD‐L1/CD45) staining, samples were incubated with PBS for 5 min followed by fixation and permeabilization with ice‐cold acetone/methanol 9:1 (v/v) for 15 min at RT. Nonspecific binding was avoided by blocking with 5% FBS in PBS at 4 °C overnight. Cells were incubated for 1 h with the anti‐CD45 mouse Ab conjugated with Alexa 647 (Santa Cruz Biotechnology). CD45 was used as a negative marker for CTCs. Cytospins were further incubated with goat anti‐PD‐L1 Ab (Novus Biologicals, Littleton, CO, USA) for 1 h followed by anti‐goat Alexa 488 secondary Ab (Life Technologies) for 45 min. A45‐B/B3 mouse Ab was used for the detection of the CK 8/18/19 (Amgen), and Alexa 555 anti‐mouse was used as a secondary Ab (Life Technologies) for 45 min. Finally, samples were mounted on Prolong antifade medium containing DAPI for nuclear visualization.

H1299 cells spiked in HDs' PBMCs were used as positive and negative controls. Negative controls were prepared by omitting one of the first Abs and incubating the cells with the respective secondary Ab. Every experiment included three different negative controls for each antibody and one positive for all the Abs. In this manner, we evaluated the sensitivity and specificity of triple immunofluorescence experiments and limited any possible crosstalk between the corresponding antibodies.

Slides were analyzed with the automated VyCAP system (VyCAP B.V., Enschede, The Netherlands). CTCs were also indicatively assessed using the open‐source accept software v1.1 (https://github.com/LeonieZ/ACCEPT, University of Twente, Enschede, The Netherlands).

CK, an epithelial marker, was used to characterize a cell as a CTC. Regarding the (CK/PD‐L1/CD45) staining, the leukocyte marker CD45 was used as a negative marker for CTC selection. In cases where CK expression was low, the cytomorphological criteria described by Meng et al. [47], such as high nuclear–cytoplasmic ratio, were also applied for CTCs' identification. Consequently, a second staining of (CK/CXCR4/JUNB) was performed, and the slides were classified according to the CTC morphology and the criteria used in the previous staining (CK/PD‐L1/CD45).

The VyCAP system is an imaging platform, where patients' slides can be scanned automatically using four different channels (DAPI, ALEXA, FITC, APC). Images for control samples and CTCs were captured based on the determined exposure time, defined by the negative and positive controls, of each antigen. The produced frames, after the automated scans, were analyzed to identify and characterize patients' CTCs. Additionally, the corresponding frames were also double‐checked using the accept software. Three different gates were applied to be able to detect all the heterogenous populations of CTCs:

1st sclc gate: CK‐ > Mean intensity > 3000 & ≤ 35 000, CK‐ > Max intensity > 15 000 & ≤ 80 000, CK‐ > Perimeter > 50 & ≤ 300, CK‐ > Size > 80 & ≤ 3000, DAPI‐ > Size ≤ 25 000.
2nd sclc gate: CK‐ > Mean intensity > 3000 & ≤ 35 000, CK‐ > Max intensity > 9500 & ≤ 80 000, CK‐ > Perimeter > 50 & ≤ 250, CK‐ > Εccentricity > 0.2 & ≤ 0.9, CK‐ > Size > 100 & ≤ 3000, DAPI‐ > Size ≤ 20 000.
3rd sclc gate: CK‐ > Mean intensity > 3000 & ≤ 35 000, CK‐ > Max intensity > 9500 & ≤ 80 000, CK‐ > Perimeter > 50 & ≤ 250, CK‐ > Εccentricity > 0.2 & ≤ 0.9, CK‐ > Size > 100 & ≤ 3000.

Finally, the identification of CTCs was performed blindly to clinical data.

### 2.4 Isolation of exosomes

Blood samples from 58 SCLC patients and 10 HD were centrifuged at 1400 ***g*** for 10 min without brakes to obtain plasma and were stored at −80 °C until use. Exosomes were isolated using the EXO‐Prep exosome precipitation kit (HansaBioMed Life Sciences, Tallinn, Estonia) according to the manufacturer's instructions. Before isolation, plasma samples were prepared by 3 centrifugations to eliminate red blood cells and cellular debris. Upon addition of the EXO‐Prep reagent to the samples in a 1/4 ratio, they were incubated on ice for 1 h. The exosome pellet was recovered by 2 consecutive centrifugations at 10 000 ***g*** for 20 min and at 1500 ***g*** for 2 min and was then resuspended in 100 μL of PBS. Exosome samples were stored at −20 °C until further use.

### 2.5 Transmission electron microscopy

Transmission electron microscopy (TEM) was used to assess the morphology of the isolated exosomes. TEM analysis was conducted on a JEOL JEM‐2100 system, operated at 200 kV (resolution: point 0.23 nm, lattice 0.14 nm). The samples were prepared by dispersion in water and spread onto a carbon‐coated copper grid (200 mesh). Samples were also negatively stained with 2% uranyl acetate in deionized water for 5 min and dried out with filter paper. TEM images were recorded by means of an Erlangshen CCD Camera (Gatan Model 782 ES500W).

### 2.6 Western blot analysis

Protein concentration was estimated using the Bradford protein assay. The exosomal protein (EP) levels were normalized to 1 mL of plasma and are reported as μg protein per mL of plasma. Exosome samples (40 μg) were analyzed by SDS/PAGE in 10% gels. Proteins were transferred onto Immobilon‐P PVDF membranes (Merck Millipore, Billerica, MA, USA) via the Trans‐Blot Turbo Transfer System (Bio‐Rad Laboratories, Hercules, CA, USA) using a custom protocol (25 V‐1.0A‐45 min). Membranes were blocked with 3% BSA in Tris‐buffered saline with 0.05% Tween 20 (TBS‐Tween 20) for 1 h at RT. Membranes were incubated overnight at 4 °C with the following primary Abs: rabbit JUNB (1 : 1000, #3753, Cell Signaling Technology), rabbit CXCR4 (1 : 1000, #ab124824, Abcam), and rabbit Syntenin‐1 (1 : 1000, #ab133267, Abcam, Cambridge, UK). Membranes were then incubated with a horseradish peroxidase‐conjugated secondary Ab (1 : 5000, #AP132P, Merck Millipore) for 1 h at RT. Bands were developed using the WesternSure PREMIUM Chemiluminescent Substrate (LI‐COR, Lincoln, NE, USA) and scanned on a C‐DiGit Blot Scanner (LI‐COR) for 12 min on high resolution. Analysis of band intensities was performed using Image Studio Digits 5.2 (LI‐COR). Syntenin‐1 has emerged as a marker for exosomes [48] and was used for normalization.

### 2.7 RNA isolation from exosomes, small RNA library preparation, miRNA sequencing, and bioinformatics analysis

Exosomes were isolated from the plasma of 2 HDs and 4 SCLC patients as described in 2.4. The pelleted exosomes were resuspended directly into TRIzol reagent (15596026, Invitrogen, Waltham, MA, USA) with one 30‐s pulse using the LABSONIC M sonicator (Sartorius AG, Göttingen, Germany) to homogenize the solutions. RNA was isolated according to the manufacturer's instructions with the addition of Linear Acrylamide (AM9520, Invitrogen) in the aqueous phase to a final concentration of 20 μg·mL^−1^. The pelleted RNA was resuspended in nuclease‐free water and analyzed using the Qubit™ RNA High Sensitivity (HS) kit (Q32852, Invitrogen) on the Qubit 4 fluorometer (Invitrogen). The pelleted RNA was resuspended in nuclease‐free water supplemented with Linear Acrylamide (AM9520, Invitrogen) to a final concentration of 20 μg·mL^−1^ and analyzed using the Qubit™ RNA High Sensitivity (HS) kit (Q32852, Invitrogen) on the Qubit 4 fluorometer (Invitrogen). Small RNA libraries were prepared with the Ion Total‐RNA Seq Kit v2 (4479789, Ion Torrent) according to the manufacturer for small RNA libraries without enrichment (Rev. B.0). Libraries were then sequenced on an Ion GeneStudio S5 System with Ion Chef (Ion Torrent, Invitrogen). The resulting fastq files were analyzed using the smallRNA plugin of the torrent suite software (v. 5.12). Briefly, reads are matched to mature miRNAs with bowtie2 included in the plugin and the featurecounts software generates miRNA raw count [49, 50]. One of the control samples failed to generate enough reads that passed quality filtering. Only miRNA counts from miRNAs that were detected in the single control sample were retained for downstream analysis. The filtered miRNA counts were uploaded to the Galaxy web platform through the usegalaxy.eu public server [51]. Differential gene expression was calculated with limma‐voom (v3.50.1) [52]. The validated targets of the top 10 miRNAs (*P*‐value), which were identified from TarBase v8, were subjected to functional enrichment analysis using the PANTHER classification platform [53, 54].

All raw sequencing data are available at the Gene Expression Omnibus (GEO) repository under the Accession Number GSE238188 (https://www.ncbi.nlm.nih.gov/geo/query/acc.cgi?acc=GSE238188 accessed on 25 July 2023).

### 2.8 Statistical analyses

Statistical tests between the mean percentages of phenotypes were carried out with Wilcoxon signed‐rank nonparametric test. Spearman's correlation coefficient was utilized to examine correlations between total CTC number per patient and CTC phenotypes in each staining. One‐way analysis of variance (ANOVA) was used to compare the protein exosomal expression in SCLC patients and HDs. ROC analysis was performed to determine the discriminative ability of JUNB and CXCR4. The correlation between protein exosomal expression and CTC phenotypes was evaluated using Spearman's correlation coefficient. PFS was defined as the period between enrollment to the study and disease relapse or death, whichever occurred first. OS was defined as the period from enrollment to the study until death from any cause or the last time follow‐up when the patient was reported alive. Kaplan–Meier analysis was used to correlate the presence of CTCs, or specific phenotypes with patients' clinical outcome, using the log‐rank test. Cox regression was also performed for the analysis of the CTCs' number per phenotype regarding patients' OS and PFS. Finally, all clinicopathological data for patients enrolled in this study were analyzed to identify any possible association with patients' outcomes. A flow chart with an outline of the study from initial recruitment to final analysis is depicted in Fig. S1. Some patients did not have available plasma samples. Other patients left the study after the blood draw; therefore, no follow‐up clinical data were available for these patients. Statistical analysis was performed using the ibm spss statistics (Version 27.0: IBM Corp, Armonk, NY, USA) and graphpad prism (Version 8.0.1, GraphPad Software, San Diego, CA, USA) softwares. The threshold for significant differences and associations was set at *P* < 0.05.

## 3 Results

### 3.1 Biomarker expression in SCLC patients' CTCs

#### 3.1.1 JUNB, CXCR4, and PD‐L1 expression in CTCs of SCLC patients

All patients were analyzed before the initiation of 1^st^‐line chemotherapy (at baseline) to avoid alterations in CTCs' phenotypes induced by chemotherapy (Table 1).

**Fig. 1:**
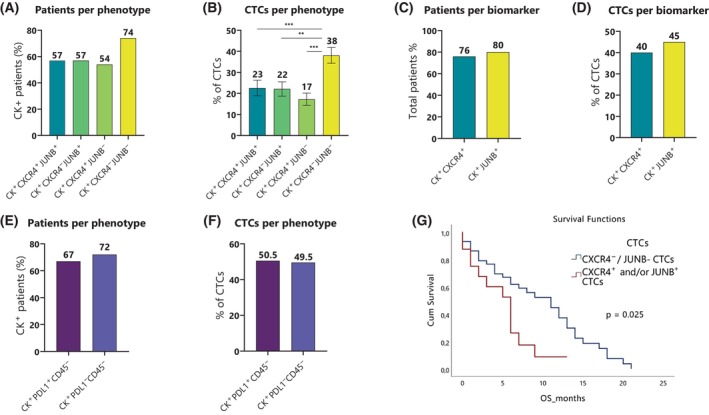
Phenotypic patterns of biomarker expression in small‐cell lung cancer (SCLC) patients. (A) Percentages of cytokeratin‐positive (CK^+^) patients with (CK^+^CXCR4^+^JUNB^+^), (CK^+^CXCR4^−^JUNB^+^), (CK^+^CXCR4^+^JUNB^−^), and (CK^+^CXCR4^−^JUNB^−^) phenotypes. (B) Mean percentages of total identified circulating tumor cells (CTCs) expressing the (CK^+^CXCR4^+^JUNB^+^), (CK^+^CXCR4^−^JUNB^+^), (CK^+^CXCR4^+^JUNB^−^), and (CK^+^CXCR4^−^JUNB^−^) phenotypes. Error bars represent the standard error of the mean (SEM). Statistical significance was determined using the Wilcoxon test, with significance indicated as follows: **P ≤ 0.01 and ***P ≤ 0.001. (C) Percentages of cytokeratin‐positive (CK^+^) patients with (CK^+^CXCR4^+^) and (CK^+^JUNB^+^) phenotypes. (D) Mean percentages of total identified circulating tumor cells (CTCs) with the (CK^+^/CXCR4^+^) and (CK^+^/JUNB^+^) phenotypes. (E) Percentages of cytokeratin‐positive (CK^+^) patients with (CK^+^PD‐L1^+^CD45^−^) and (CK^+^PD‐L1^−^CD45^−^) phenotypes. (F) Mean percentages of total identified circulating tumor cells (CTCs) expressing the (CK^+^PD‐L1^+^CD45^−^) and (CK^+^PD‐L1^−^CD45^−^) phenotypes. (G) Kaplan–Meier survival curve revealing that extended‐stage SCLC patients expressing CXCR4 and/or JUNB in their circulating tumor cells (CTCs) exhibited lower overall survival (OS) compared to patients expressing CK^+^CXCR4^−^JUNB^−^ CTCs (*P* = 0.025). Statistical significance was determined using the log‐rank test.

In the (CK/CXCR4/JUNB) staining, CTCs were detected in 70% of patients (70 out of 100). Among the CK‐positive SCLC patients, 57% (40 out of 70) had the (CK^+^CXCR4^+^JUNB^+^) phenotype and similarly 57% (40 out of 70) had the (CK^+^CXCR4^−^JUNB^+^) phenotype. The (CK^+^CXCR4^+^JUNB^−^) phenotype was observed in 54% (38 out of 70), and the (CK^+^CXCR4^−^JUNB^−^) phenotype was detected in 74% (52 out of 70) of patients (Fig. 1A). Chi‐square analysis did not show a statistically significant difference regarding the percentage of patients belonging to different subgroups (Fig. 1A). Moreover, 24% of patients (17 out of 70) revealed only one of the four identified phenotypes, 34% (24 out of 70) harbored two, 17% (12 out of the 70) had three, and 24% (17 out of 70) harbored all the examined phenotypes (Table S1).

Regarding the frequency of phenotypes among the total identified CTCs, 23% of the CTCs were classified as (CK^+^CXCR4^+^JUNB^+^), 22% as (CK^+^CXCR4^−^JUNB^+^), 17% as (CK^+^CXCR4^+^JUNB^−^), and 38% were characterized as (CK^+^CXCR4^−^JUNB^−^). The frequency of (CK^+^CXCR4^−^JUNB^−^) CTCs was significantly higher compared to all the other phenotypes [(CK^+^CXCR4^+^JUNB^+^): Wilcoxon test: *P* < 0.001, (CK^+^CXCR4^−^JUNB^+^): *P* = 0.002 and (CK^+^CXCR4^+^JUNB^−^): *P* < 0.001; Fig. 1B].

Considering CXCR4 and JUNB alone, 76% of the CK^+^ patients (53 out of 70) were CXCR4‐positive and 80% (56 out of 70) were JUNB‐positive (Fig. 1C). The mean percentage of CXCR4‐positive and JUNB‐positive CTCs was 40% and 45%, respectively (Fig. 1D). Additionally, Spearman's correlation coefficient revealed a positive correlation between the total CTC number per patient and each of the four observed CTC phenotypes (Table S2).

In the (CK/PD‐L1/CD45) staining, CTCs were detected in 57% of patients (57 out of 100). Among the CK‐positive SCLC patients, 67% (38 out of 57) had the (CK^+^PD‐L1^+^CD45^−^) phenotype and the most frequent phenotype was (CK^+^PD‐L1^−^CD45^−^) with 72% (41 out of 57) (Fig. 1E). Moreover, 61% of patients (35 out of 57) possessed one of the identified phenotypes, while both phenotypes were detected in 39% (22 out of 57) (Table S3). Regarding the mean percentages of the observed phenotypes in total identified CTCs, 50.5%were classified as (CK^+^PD‐L1^+^CD45^−^) and 49.5% were characterized as (CK^+^PD‐L1^−^CD45^−^), with no statistical significance found regarding phenotype frequency (Fig. 1F). Moreover, the total CTC number per patient positively correlated with the two observed CTC phenotypes (Table S4). Lastly, Spearman analysis revealed that CTCs expressing PD‐L1 correlated with all CTC phenotypes expressing JUNB and/or CXCR4 (*P <* 0.001).

**Fig. 2:**
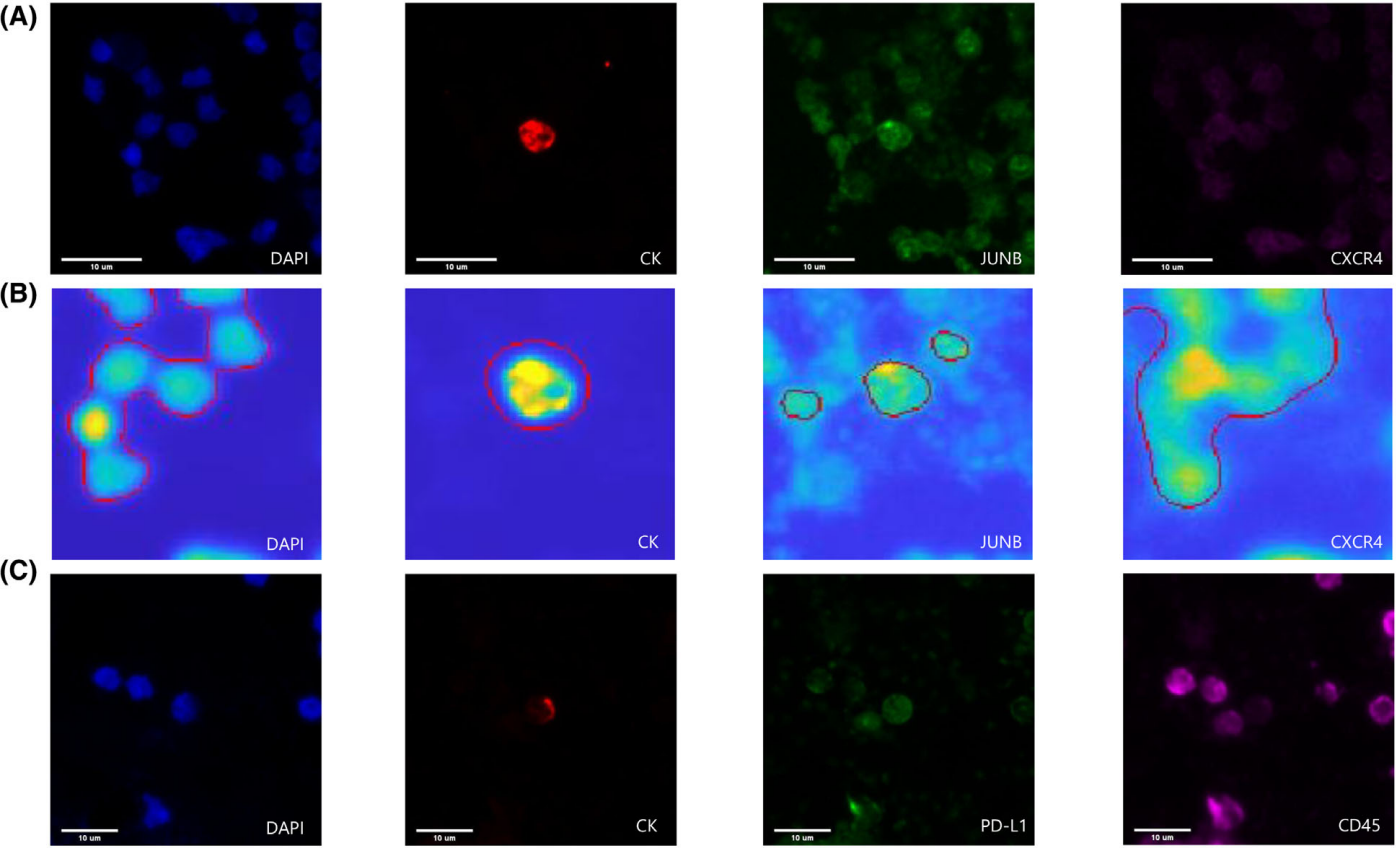
Circulating tumor cell (CTC) isolated from a small‐cell lung cancer (SCLC) patient. (A) CTCs stained with cytokeratin (CK) (red), JUNB (green), and CXCR4 (purple). Nuclei (blue) were stained with DAPI. Image acquired from the VyCAP system (Magnification 40×). Scale bars represent 10 μm. (B) Identification of a SCLC CTC using the open source accept software. The red contours around the objects indicate the potential cells of interest as detected by the ACCEPT image analysis algorithm. Scale bars represent 10 μm. (C) CTCs stained with cytokeratin (CK) (red), PD‐L1 (green), and CD45 (purple). Nuclei (blue) were stained with DAPI. Image acquired from the VyCAP system (Magnification 40×). Scale bars represent 10 μm.

The expression of JUNB and CXCR4 in SCLC patients' CTCs is shown in Fig. 2A. Slides were scanned by the VyCAP platform. ACCEPT software (Fig. 2B) was indicatively used to automatically identify cancer cells, providing simultaneous information regarding the expression level (intensity) of JUNB and CXCR4. PD‐L1's expression is illustrated in Fig. 2C.

#### 3.1.2 Clinical significance in CTCs

Clinical data for PFS and OS were available for 80 and 93 of the total 100 SCLC patients, respectively. Regarding disease stage, clinical data were available for 90 patients, with 77 (86%) being classified as extensive disease. Interestingly in this subgroup, patients harboring CXCR4‐ and/or JUNB‐positive CTCs revealed worse OS compared to patients without CXCR4 and JUNB expression their CTCs [4.99 months (range 3.04–6.93) vs 9.58 months (range 7.52–11.63) respectively; *P* = 0.025, HR = 2.14 (95% CI: 1.06–4.33); Fig. 1G]. Cox regression analysis revealed also a significant difference in OS (*P =* 0.021). However, survival analysis of the total number of CTCs (OS: *P =* 0.397 and for PFS: *P =* 0.586), the rest of CTCs phenotypes, and the patients' clinicopathological characteristics did not reveal any statistically significant results. Cox regression multivariate analysis did not reveal an independent prognostic factor.

No significant differences regarding PFS and OS were observed in survival analyses between patients' subgroups treated with different first‐line therapies.

### 3.2 Exosomal analysis in SCLC patients

#### 3.2.1 Characterization of exosomes isolated from the plasma of SCLC patients

**Fig. 3:**
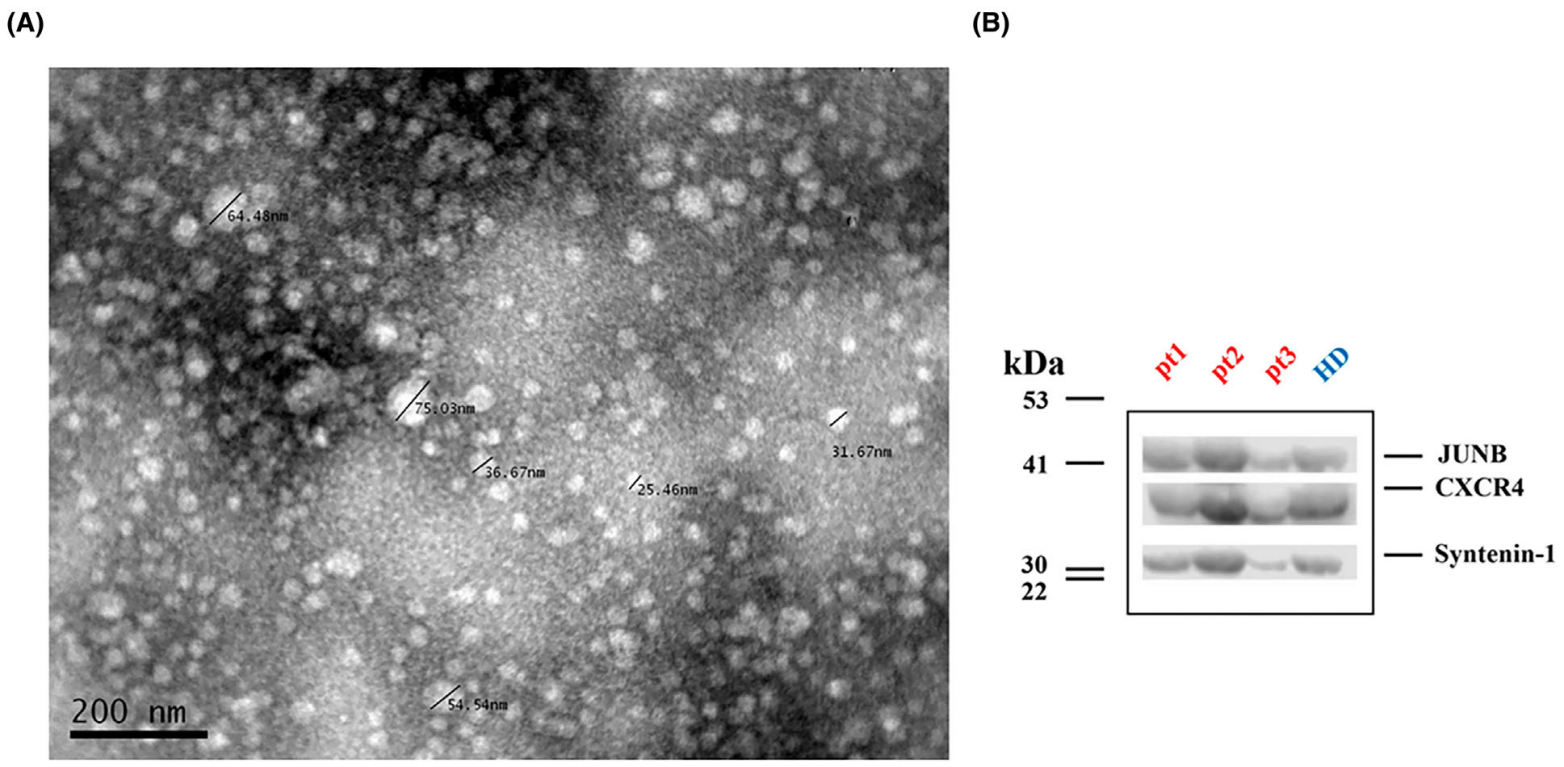
Characterization of exosomes isolated from small‐cell lung cancer (SCLC) patient plasma. (A) Transmission electron microscopy (TEM) image of isolated exosomes. Scale bar represents 200 nm. (B) Representative blots depicting JUNB and CXCR4 protein exosomal expression in exosomes isolated from 3 SCLC patients and 1 healthy donor (HD). Syntenin‐1 was used for normalization. Each western blot (WB) lane contained 40 μg exosomal protein.

Consequently, the biomarkers with clinical significance (JUNB and CXCR4) for SCLC patients were evaluated in patients' exosomes. Size characterization of exosomes was performed indicatively on one SCLC patient's sample to evaluate the quality of the exosomes and the assay efficacy by transmission electron microscopy (TEM). Regarding the isolated EVs, no contamination with other cellular fragments was observed in the samples, under TEM analysis. Isolated EVs ranged from 30 to 75 nm (Fig. 3A). Western blots verified that the isolated EVs carried Syntenin‐1, an abundant protein, implicated in exosome biogenesis [48] (Fig. 3B).

Subsequently, exosomes were isolated from the plasma of 58 SCLC patients at baseline and 10 HDs. Protein expressions of JUNB and CXCR4 were assessed by western blot, while Syntenin‐1 served as control of the exosomal origin. An equal protein amount was loaded from every sample. Figure 3B indicates the presence of JUNB and CXCR4 in exosomes, from the plasma of 3 SCLC patients relative to an HD. Protein exosomal expression of JUNB and CXCR4 was normalized by calculating the ratio of JUNB/Syntenin‐1 or CXCR4/Syntenin‐1 signal values after quantification in Image Studio Digits Ver 5.2 (LI‐COR), as shown in Fig. S2.

#### 3.2.2 Patient JUNB and CXCR4 expression compared to healthy donors

One‐way ANOVA analysis revealed a significant difference between JUNB protein exosomal expression in SCLC patients compared to HDs (*P* = 0.003; Fig. S3A). Receiver operating characteristic (ROC) analysis regarding JUNB expression in SCLC patients compared to HDs yielded a c‐statistic of 0.859 (95% CI: 0.763–0.955) (Fig. S3B), indicating a high discriminative ability.

Similarly, CXCR4 protein exosomal expression was significantly different in SCLC patients in comparison with HDs (*P* = 0.027; Fig. S3C). ROC analysis produced a c‐statistic of 0.794 (95% CI: 0.671–0.917; Fig. S3D), depicting a good discriminative ability among SCLC patients and HDs.

**Fig. 4:**
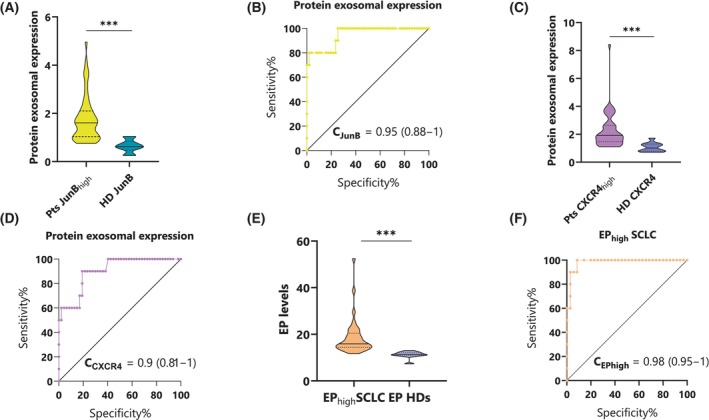
Comparison of exosomal JUNB and CXCR4 protein overexpression and total exosomal protein (EP) levels in plasma exosomes of small‐cell lung cancer (SCLC) patients versus healthy donors (HDs). (A) Protein exosomal expression of JUNB. Data are represented as violin plot with the solid line indicating the median and the dotted lines representing the interquartile range. The *P*‐value was calculated using one‐way analysis of variance (ANOVA), with significance indicated as follows: ***P ≤ 0.001. (B) Receiver operating characteristic (ROC) curve for JUNB protein exosomal expression. (C) Protein exosomal expression of CXCR4. Data are represented as violin plot with the solid line indicating the median and the dotted lines representing the interquartile range. The *P*‐value was calculated using one‐way ANOVA, with significance indicated as follows: ***P ≤ 0.001. (D) ROC curve for CXCR4 protein exosomal expression. (E) Exosomal protein overexpression (EPhigh) levels in SCLC patients compared to EP levels in HDs. Data are represented as violin plot with the solid line indicating the median and the dotted lines representing the interquartile range. The *P*‐value was calculated using one‐way analysis of variance (ANOVA), with significance indicated as follows: ***P ≤ 0.001. (F) ROC curve for the exosomal protein overexpression (EPhigh) levels in SCLC patients compared to EP levels in HDs.

Consequently, patients were categorized based on their JUNB and CXCR4 exosomal expression, setting as a threshold, the median value of the biomarkers' expression in HDs (median_JUNB_ = 0.62; median_CXCR4_ = 1.01). Specifically, patients were divided into high (≥ 0.62) and low (< 0.62) ‘expression’ for JUNB (JUNB_high_ and JUNB_low_) and high (≥ 1.01) and low (< 1.01) expression regarding CXCR4 (CXCR4_high_ and CXCR4_low_). Fifty‐one out of the 58 patients (88%) were classified as JUNB_high_, while 47 out of 58 patients (81%) were classified as CXCR4_high_. One‐way ANOVA analysis depicted a significant difference when comparing the protein exosomal expression in JUNB_high_ (*P* = 0.001) and CXCR4_high_ (*P* = 0.005) patients to JUNB and CXCR4 expression in HDs, respectively (Fig. 4A,C). A very high discriminative ability was observed for both JUNB [c‐statistic = 0.949 (95% CI: 0.882–1)] and CXCR4 [c‐statistic = 0.903 (95% CI: 0.81–0.996)] protein exosomal overexpression in SCLC patients compared to HDs, as shown in Fig. 4B,D.

Interestingly, CXCR4 protein exosomal overexpression correlated positively with SCLC patients' CTCs and their phenotypes following Spearman analysis (Table 2).

**Table 2:** Spearman correlations between CXCR4 exosomal overexpression and circulating tumor cells (CTCs') phenotypes.

Exosomes & CTC phenotypes	rho	*P* value
CXCR4^+^ exosomes and ≥ 2 CTCs	0.297	0.024
CXCR4^+^ exosomes and CXCR4^+^ CTCs	0.342	0.009
CXCR4^+^ exosomes and ≥ 2 CK^+^CXCR4^+^JUNB^+^ CTCs	0.312	0.017
CXCR4^+^ exosomes and CK^+^CXCR4^+^JUNB^−^ CTCs	0.259	0.049

Particularly, CXCR4 exosomal overexpression in SCLC patients positively correlated with the presence of ≥ 2 CTCs (rho = 0.297, *P* = 0.024) and CXCR4^+^ CTCs (rho = 0.342, *P* = 0.009). Additionally, CXCR4 exosomal overexpression was also positively correlated with the presence of ≥ 2 (CK^+^CXCR4^+^JUNB^+^) CTCs (rho = 0.312, *P* = 0.017) and (CK^+^CXCR4^+^JUNB^−^) CTCs (rho = 0.259, *P =* 0.049).

#### 3.2.3 Exosomal protein (EP) levels in patients with SCLC

Total EP levels isolated from the plasma of SCLC patients were elevated compared to those in HDs, although no significant difference was found. ROC analysis indicated a poor discriminative ability [c‐statistic = 0.631 (95% CI: 0.5–0.76)].

However, when patients' EP levels were categorized into low (< 11.42 μg·mL^−1^) and high (≥ 11.42 μg·mL^−1^), based on the median value of EP in HDs, a significant difference was found when comparing high EP levels to EP levels in HDs (*P* = 0.005; Fig. 4E). Notably, high EP levels displayed an excellent discriminative ability [c‐statistic = 0.98 (95% CI: 0.95–1); Fig. 4F].

### 3.3 Differential expression and functional impact of exosome‐derived miRNAs

**Fig. 5:**
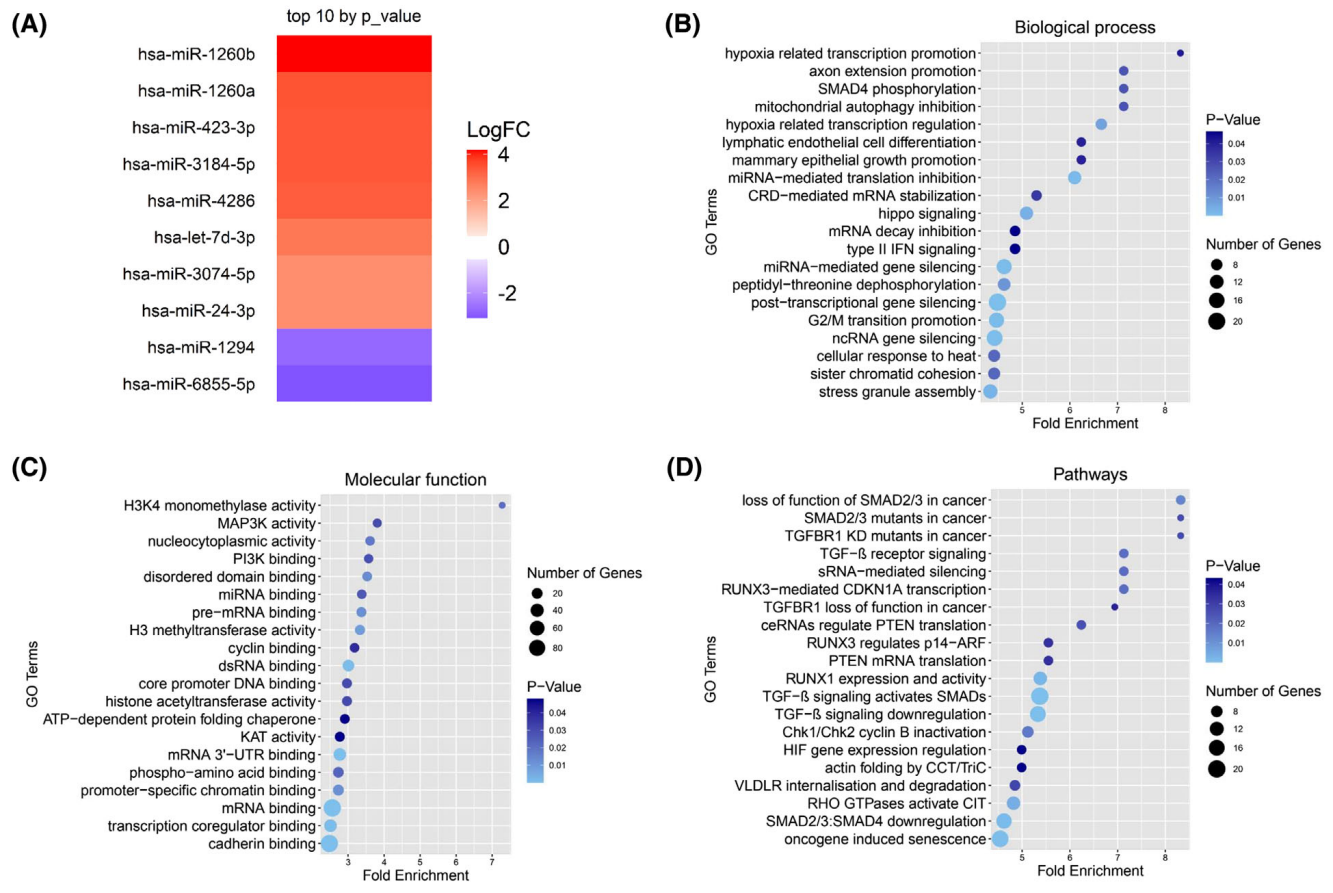
Exosomal microRNAs (miRNAs) and their validated targets. (A) Heatmap indicating the top 10 miRNAs (by *P*‐value), out of which the validated targets were analyzed. (B) Gene Ontology (GO) biological processes of the exosomal microRNA (exoMIR) validated targets. (C) Gene Ontology (GO) molecular function analysis of the exosomal microRNA (exoMIR) validated targets. (D) Reactome pathways of the exosomal microRNA (exoMIR) validated targets.

The crucial involvement of SCLC‐derived exomes was explored in the present study to investigate their possible modulatory role in various biological processes and signaling pathways that are linked to tumorigenesis and metastasis. In addition, specific observed exoMIR profiles could have significant diagnostic value for patients, in association with the protein factors under study. Our analysis identified 8 miRNAs exhibiting an increased expression in the exosomes of SCLC patients, including hsa‐miR‐1260b, hsa‐miR‐4286, hsa‐miR‐3184‐5p, hsa‐miR‐423‐3p, hsa‐miR‐1260a, hsa‐let‐7d‐3p, hsa‐miR‐24‐3p, and hsa‐miR‐3074‐5p (Fig. 5A; Table S5). Conversely, hsa‐miR‐6855‐5p and hsa‐miR‐1294 were observed to have a decrease in expression levels (Fig. 5A). Notably, the upregulated hsa‐miR‐423‐3p and hsa‐miR‐4286 were found to target *JUNB* and *FOS*, as well as *FOSL2* and *ATF‐6B* genes, respectively, as identified from TarBase v8 [53] (Table S6).

Validated targets of the top 10 deregulated exoMIRs were submitted to enrichment analyses, including the Gene Ontology (GO) biological processes [55]. In addition to their role in regulating protein transport and localization, targets of these exoMIRs have been observed to participate in intricate cellular processes such as the modulation of transcription from RNA polymerase II promoter under hypoxic conditions, the inhibition of mitochondrial autophagy, and the silencing of genes through miRNA‐mediated translation inhibition (Fig. 5B). GO molecular function analysis further showed that the above miRNA targets have the potential to influence a range of cellular functions, including but not limited to histone methylation and MAP kinase activity (Fig. 5C). Additional enriched pathways refer to RNA metabolism and transcription, highlighting thus the diverse mechanisms through which they may contribute to the onset or progression of disease. Finally, enrichment analysis in the Reactome pathways database [56] reported the modulation of several cancer‐related pathways, including the transforming growth factor‐beta (TGF‐β) and hypoxia‐inducible factor (HIF) signaling pathways (Fig. 5D).

### 3.4 Clinical relevance in SCLC patients' exosomes

**Fig. 6:**
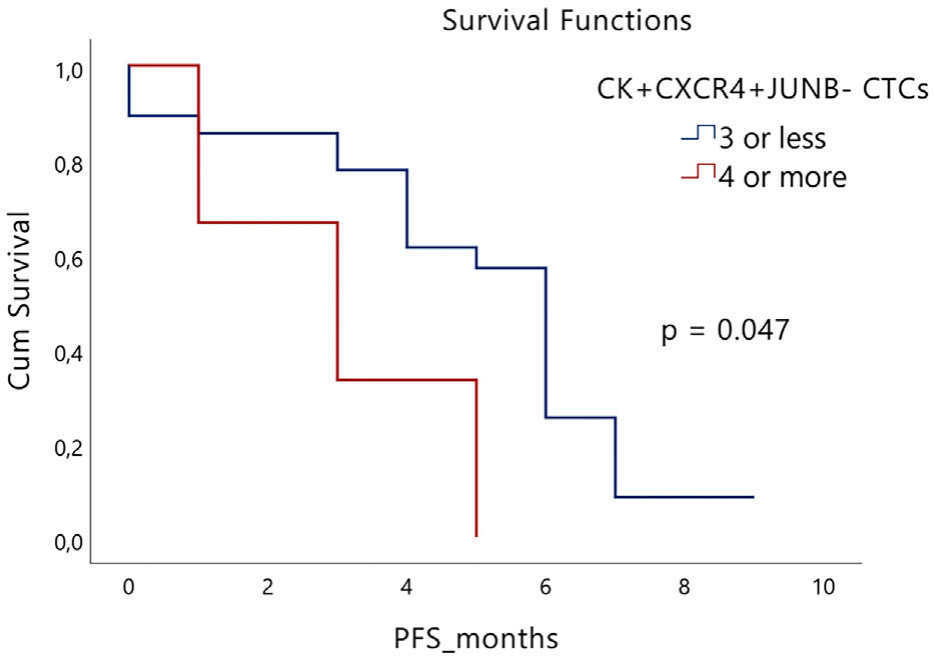
Clinical significance of CXCR4 protein exosomal expression in small‐cell lung cancer (SCLC) patients. Kaplan–Meier survival curve depicting that patients with CXCR4 protein exosomal overexpression showcased a significantly shorter progression‐free survival (PFS) when harboring ≥ 4 CK^+^CXCR4^+^JUNB^−^ circulating tumor cells (CTCs) compared to 3 or less CK^+^CXCR4^+^JUNB^−^ CTCs (*P* = 0.047). Statistical significance was determined using the log‐rank test.

Clinical data for PFS and OS were available for 40 and 52 of the 58 patients, in which exosomal analysis was performed. Combined analysis of exosomal protein expression and patients' CTCs revealed that the subgroup of SCLC patients with CXCR4 exosomal overexpression (47 out of 58), who harbored four or more CTCs with the (CK^+^CXCR4^+^JUNB^−^) phenotype, exhibited lower PFS [3 months (range 0.74–5.26) vs 4.99 months (range 4.01–5.97), respectively; *P* = 0.047, HR = 0.31 (95% CI: 0.09–1.11); Fig. 6], compared to patients that had three or less (CK^+^CXCR4^+^JUNB^−^) CTCs. Survival analyses for the rest observed phenotypes regarding CXCR4 and JUNB protein exosomal expression and patients' clinical data (PFS and OS) did not reveal any statistically significant results. Cox regression multivariate analysis for the observed CTC phenotypes and patients' clinicopathological characteristics (Table 1) did not reveal an independent prognostic factor.

## 4 Discussion

The high mortality of SCLC coupled with limited therapeutic options elucidates the necessity for new biomarkers, companion diagnostic tests, and innovative treatment approaches, in addressing this severe disease. In this study, we evaluated the expression of JUNB, CXCR4, and PD‐L1 in SCLC patients' CTCs. We further assessed the potential diagnostic and prognostic value of these molecules in CTCs and in patients' exosomes.

CTCs are a poor prognostic factor for SCLC patients [57, 58, 59, 60]. However, their detection and characterization remain challenging due to a lack of epithelial characteristics [11] and high heterogeneity [61, 62]. Conversely, EVs can be isolated from all patients' samples [63]. However, their prognostic role in SCLC is still under investigation [36]. Simultaneous analysis of liquid biopsy approaches CTCs, exosomes [64, 65], and ctDNA [9, 66, 67] can provide a comprehensive real‐time description of the disease state. Thus, this study provides robust evidence connecting JUNB and CXCR4 expression in CTCs and exosomes (Table 2) documenting their prognostic (Figs 1G and 6) and potential diagnostic (Fig. 4 and Fig. S3) significance.

Our data showed significant inter‐ and intrapatient heterogeneity regarding CTCs' subpopulations; however, all the examined biomarkers were expressed in most cases (Fig. 1, Tables S1 and S3). Our findings on JUNB and CXCR4 verify our prior studies on breast cancer‐derived CTCs [27] and DTCs [28]. A previous study from our group, involving a smaller cohort of lung cancer patients, revealed similar biomarker expression levels, indicating that elevated expression of CXCR4 and JUNB is associated with poorer OS in extended‐stage SCLC [29]. This larger study, encompassing one hundred SCLC patients, further substantiates the prognostic significance of CXCR4‐positive CTCs and reveals that JUNB also plays a significant role. Patients harboring CXCR4^+^ and/or JUNB^+^ CTCs had poorer OS (*P* = 0.025) compared to those with the double‐negative phenotype (CK^+^CXCR4^−^JUNB^−^, Fig. 1G). These findings underscore the importance of CXCR4 and JUNB as potential prognostic SCLC biomarkers, in line with previous research linking CXCR4 overexpression to metastasis regulation [68, 69] and to prognostic significance regarding PFS [70] in SCLC patients. Analysis of the total CTCs number did not show any significant results regarding OS or PFS. This could be attributed either to the technique used in the current study or to the fact that the total number of CTCs precludes a very heterogeneous population of cells. Some of them could have a prognostic significance; however, others are destined to die as we have shown in previous studies [15, 71, 72, 73]. Therefore, it is possible that a specific phenotype can have more prognostic power compared to the total number of CTCs. This is something we have observed in previous studies in breast [27], NSCLC, and SCLC [29] patients. Therefore, although CTCs' enumeration often provides statistically significant results regarding OS and/or PFS, this is not the case in all the studies with a relatively small number of patients. The absence of clinical relevance in the total number of CTCs was also observed in studies with comparable patient numbers from other groups and for different types of cancers, even using the CellSearch system [74].

PD‐L1 is frequently overexpressed in CTCs and is potentially associated with clinical outcomes, serving as a target for personalized immunotherapy [6]. However, in SCLC, studies have reported inconsistent findings regarding PD‐L1 expression in tumor tissues and CTCs, with both high [75] and low [76, 77] expression levels observed. In our cohort, the mean percentage of PD‐L1‐positive CTCs was 50% (Fig. 1F). Although no correlation was found between PD‐L1 expression and patient survival, Spearman analysis showed a significant correlation among CTCs expressing PD‐L1, JUNB, and/or CXCR4 (*P* < 0.001). This suggests a shared pathway for these biomarkers, supported by previous studies. Green et al. [26] found that JUNB binds to the PD‐L1 promoter, promoting its transcription, while Sfakianakis et al. [78] identified a link between JUNB and CXCR4. Additionally, a recent study reported elevated PD‐L1 levels in microglia subpopulations overexpressing JUNB, which regulate melanoma brain‐metastasis progression [79].

Exosomes represent the most investigated subgroup of EVs, with the understanding of their intercellular exchanges and underlying molecular mechanisms being the subject of extensive research. [80]. Tumor‐derived exosomes portray an accessible and efficient avenue, bearing several notable advantages, including their substantial presence in bodily fluids, coupled with their potential as cancer biomarkers due to the assortment of proteins, lipids, and miRNAs within their cargo [81, 82]. Exosome isolation was conducted on 58 of 100 SCLC patients. JUNB and CXCR4 protein levels in exosomes were significantly elevated in patients compared to healthy donors (HDs) (Fig. S3A,C). Both proteins exhibited strong discriminative potential (Fig. S3B,D), particularly when the median exosomal expression value of HDs was employed as a threshold. The discriminative potential of JUNB and CXCR4 was notably high (95% and 90%, respectively) (Fig. 4B,D), underscoring their viability as potential diagnostic biomarkers. Furthermore, total protein levels in SCLC‐derived plasma exosomes were significantly higher compared to HDs (Fig. 4E), in line with previous findings by Vetsika et al. [83] in NSCLC patients. Elevated EP levels demonstrated excellent discriminative ability (Fig. 4F), reinforcing their potential utility as a diagnostic tool for lung cancer. Remarkably, the (CK^+^CXCR4^+^JUNB^−^) CTC phenotype was clinically relevant in patients overexpressing CXCR4 in their exosomes, as we showcased that in this subcohort, the presence of four or more (CK^+^CXCR4^+^JUNB^−^) CTCs was associated with shorter PFS compared to patients harboring three or less CTCs expressing that phenotype (Fig. 6). This finding bolsters the notion that the combination of liquid biopsy components, such as CTCs and exosomes, holds the potential for improved utility in advancing patient prognosis and diagnostic accuracy.

Complementary analyses included next‐generation sequencing of exoMIRs from HDs and SCLC patients to identify specific miRNAs implicated in SCLC pathogenesis and progression. Several miRNAs with distinct roles in cancer were identified, including hsa‐miR‐1260b [84, 85], hsa‐miR‐4286 [86], and hsa‐miR‐3184‐5p [87], which exhibited dysregulation in SCLC‐derived exosomes (Fig. 5A). Notably, hsa‐miR‐423‐3p and hsa‐miR‐4286 were upregulated and are known to target cancer‐related genes [88, 89] such as JUNB and members of the AP‐1 family (FOS, FOSL2, ATF‐6B). These findings suggest a potential relationship between AP‐1 family genes and SCLC exoMIRs (Table S6) [30, 31], underscoring their potential as prognostic biomarkers or therapeutic targets. Enrichment analyses revealed significant pathways involved in cancer pathogenesis, particularly the TGF‐β pathway (Fig. 5B–D). Given its frequent association with cancer [90, 91], as well as regulating JUNB [92], further investigation of the identified miRNAs is warranted. Targeting these miRNAs might modulate their expression and disrupt oncogenic pathways, potentially inhibiting cancer progression. Additionally, analyzing miRNA expression patterns could facilitate the development of noninvasive diagnostic tools and prognostic biomarkers, thus enhancing patient care and clinical outcomes in SCLC.

Our study reveals significant overexpression of JUNB and CXCR4 in SCLC CTCs, indicating their strong discriminative potential based on their protein exosomal expression, compared to HDs. These findings enhance our understanding of SCLC's molecular landscape and lay the foundational groundwork for clinical applications. The concurrent expression of JUNB and CXCR4 demonstrates prognostic significance (Fig. 1G), suggesting a basis for developing prognostic assays and diagnostic methods for early SCLC detection, utilizing an accessible and affordable approach. CTCs provide direct insights into metastatic potential, while exosomes offer a rich source of biomolecules reflecting SCLC's molecular profile. However, this study has limitations, including the low recovery rate of CTCs using the Ficoll density gradient centrifugation method and uneven patient numbers for CTCs and exosomal analyses (100 and 58, respectively). These discrepancies can strongly impact survival analyses and correlations between CTCs and exosomes. Further investigation in larger patient cohorts, as well as other cancer types, is essential to validate the prognostic and diagnostic significance of JUNB, CXCR4, and PD‐L1 in both CTCs and exosomes. Ongoing research is also exploring the regulatory roles of specific SCLC‐derived exoMIRs, elucidating their impact on disease progression and metastasis.

## 5 Conclusions

JUNB, CXCR4, and PD‐L1 were all found overexpressed in CTCs, with the presence of CXCR4 and/or JUNB being associated with their clinical outcome. Additionally, the latter biomarkers indicated a high discriminative ability when expressed in exosomes compared to HDs. The profile of exoMIRs identified in this study revealed important putative miRNA targets, suggesting their involvement in SCLC onset and progression. More importantly, this is the first study demonstrating the concurrent utilization of two liquid biopsy components (CTCs and exosomes) from SCLC patients, to establish this approach as a valuable prognostic and/or diagnostic tool for the disease.

## Conflict of interest

The authors declare no conflict of interest.

## Author contributions

Conceptualization, GK; methodology, GK, and EP, AR, DP, CS; validation, DP, AR; formal analysis, DP, and AR; investigation, GK, CS; resources, AC, FK, EC, AC, AnK, F‐ID, AX, AtK, EC and VG; data curation, DP, AR, A‐NS, CS, AC, AnK, F‐ID, EC, FK, AX, AtK; writing—original draft preparation, DP, AR, A‐NS, CS, and GK; writing—review and editing, DP, AR, A‐NS, CS, AC, AnK, F‐ID, EC, FK, AX, AtK and VG; visualization, VG, GK; supervision, GK, CS; project administration, GK; funding acquisition, GK.

## Peer review

The peer review history for this article is available at https://www.webofscience.com/api/gateway/wos/peer‐review/10.1002/1878‐0261.13765.

## Supporting information


**Fig. S1.** Flow chart of Small Cell Lung Cancer (SCLC) patients, depicting the participants enrolled in the study, as well as each stage of study design.

**Fig. S2.** Western Blot image acquired from Image Studio Digits Ver 5.2 (LI‐COR), which was utilized for the signal values quantification.

**Fig. S3.** JUNB and CXCR4 protein exosomal expression comparison between Small Cell Lung Cancer (SCLC) patients' and Healthy Donors (HDs') plasma exosomes.

**Table S1.** Number of Circulating Tumor Cells (CTCs) per phenotype and per patient, regarding CXCR4 and JUNB expression.

**Table S2.** Correlation between total Circulating Tumor Cells (CTCs) count per patient and the examined CTCs phenotypes regarding CXCR4 and JUNB.

**Table S3.** Number of Circulating Tumor Cells (CTCs) per phenotype and per patient, regarding Programmed death‐ligand 1 (PD‐L1) expression.

**Table S4.** Correlation between total Circulating Tumor Cells (CTCs) count per patient and the examined CTC phenotypes regarding Programmed death‐ligand 1 (PD‐L1) expression.

**Table S5.** Differential expression analysis of Small Cell Lung Cancer (SCLC)‐derived exosomal MicroRNAs (miRNAs).

**Table S6.** Validated exosomal MicroRNA (miRNA) targets as identified from TarBase v8.

## Data Availability

Data presented in the study are available upon request from the corresponding author.
