# Supplementary figures and images for: Integrative analysis of circulating tumor cells (CTCs) and exosomes from small‐cell lung cancer (SCLC) patients: a comprehensive approach
Source: Mol Oncol. 2024 Nov 22;19(7):2038–55. doi: 10.1002/1878-0261.13765 (PMC12234381; doi:10.1002/1878-0261.13765)

**Supplementary Figure 1.**

**
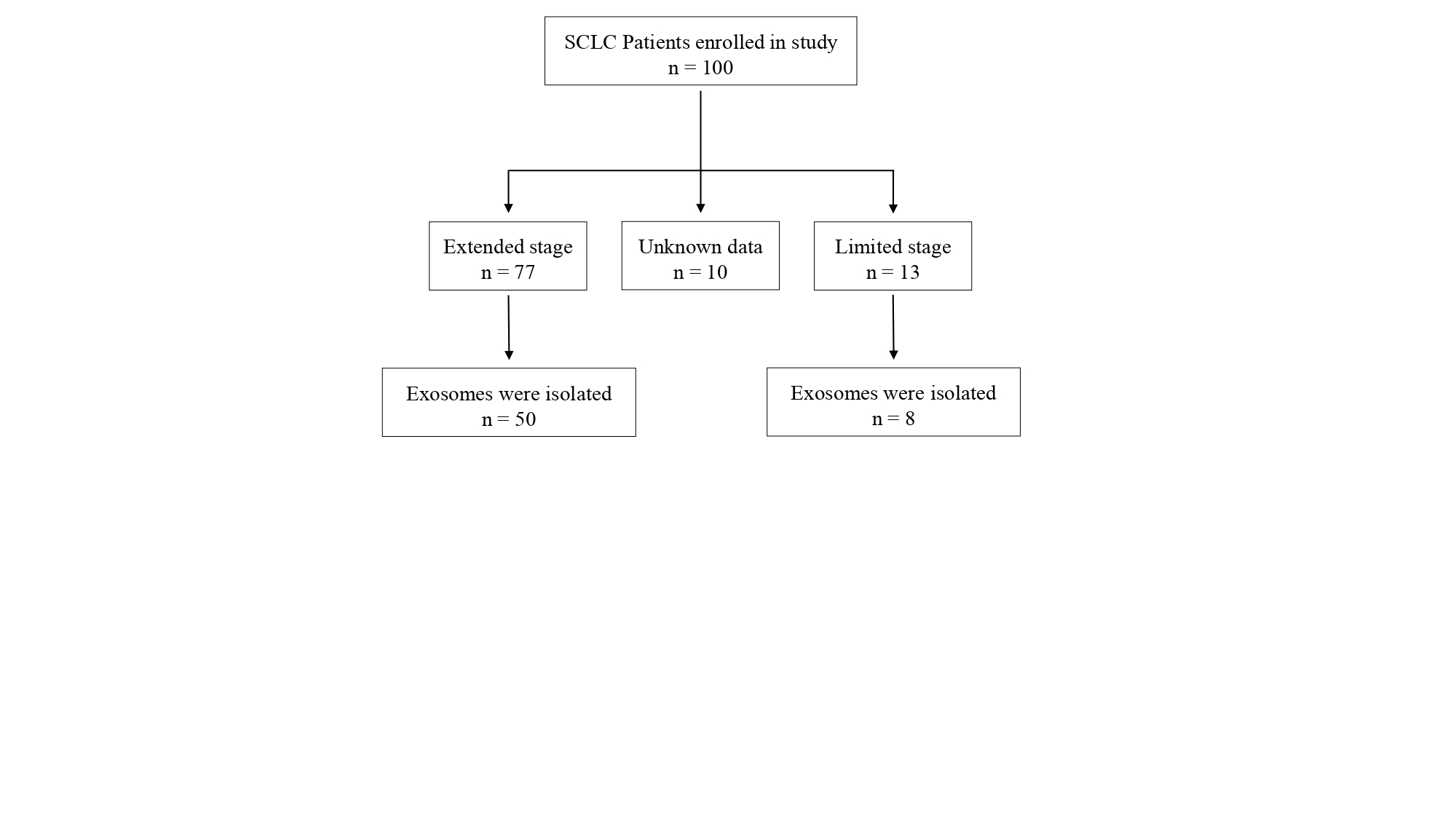
**

Supplement: Supplementary file 1 — Fig. S1. Flow chart of Small Cell Lung Cancer (SCLC) patients, depicting the participants enrolled in the study, as well as each stage of study design. [file MOL2-19-2038-s003.docx]

**Supplementary Figure 2.**

**
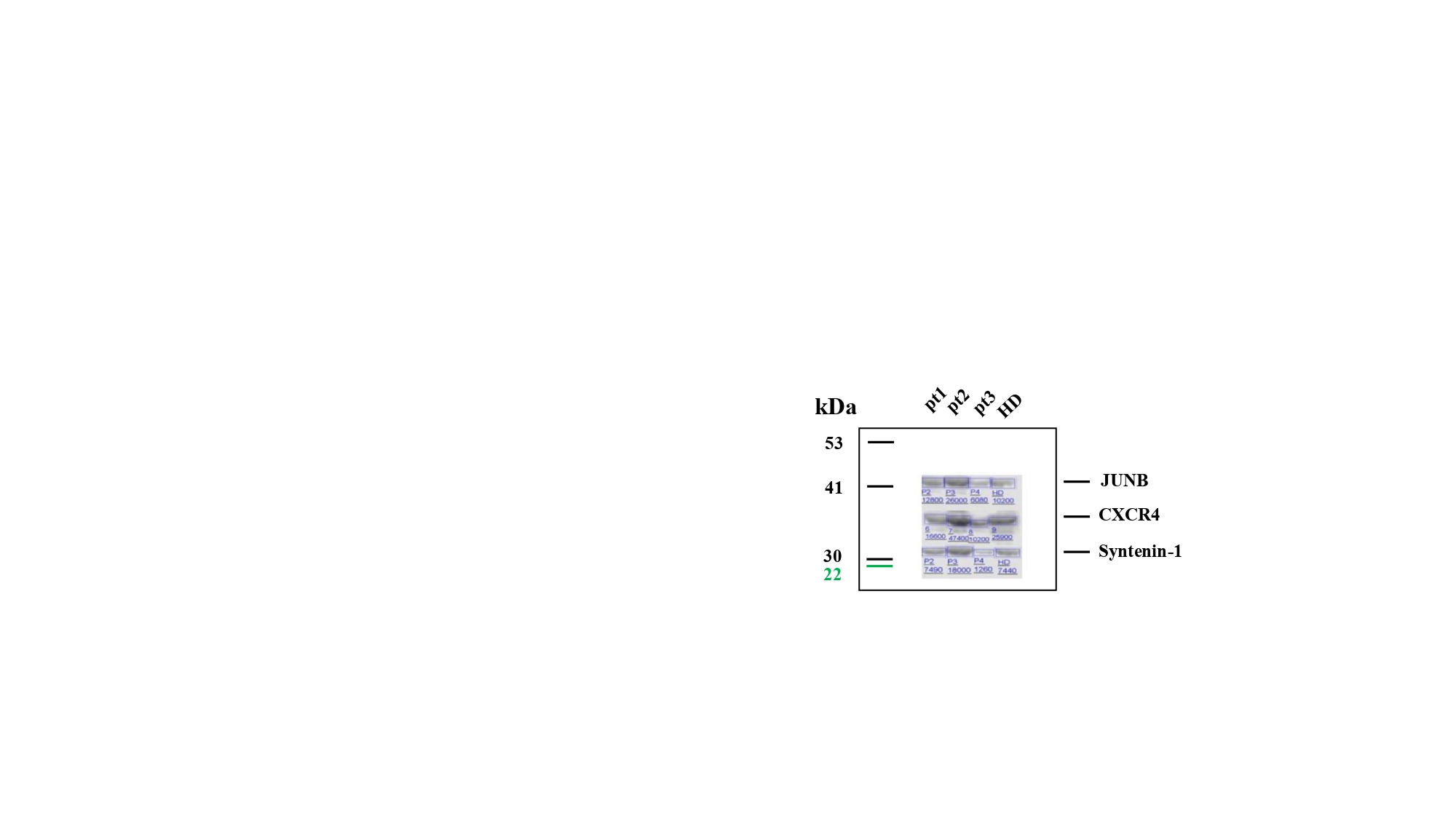
**

Supplement: Supplementary file 2 — Fig. S2. Western Blot image acquired from Image Studio Digits Ver 5.2 (LI‐COR), which was utilized for the signal values quantification. [file MOL2-19-2038-s004.docx]

**Supplementary Figure 3.**

**
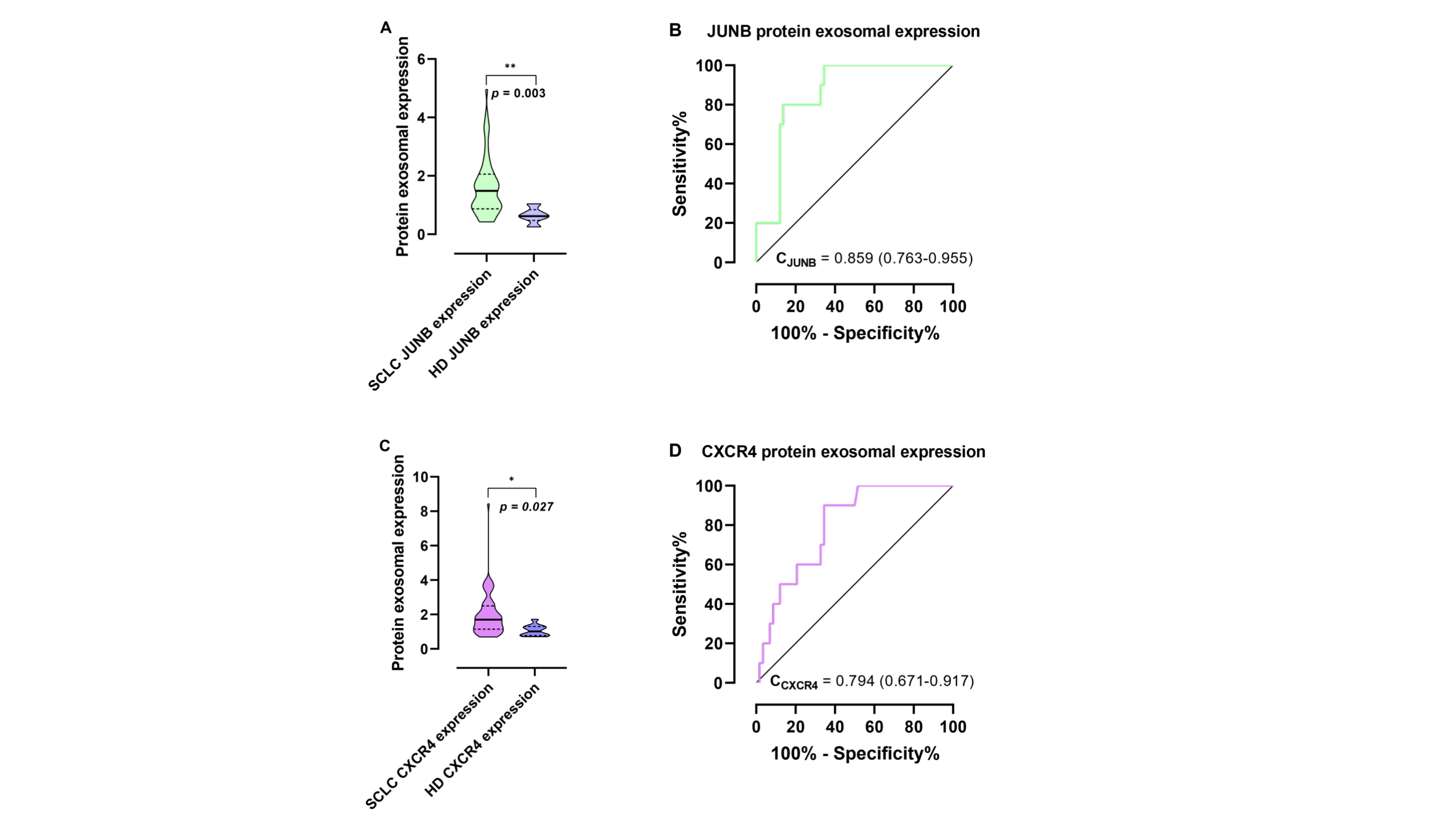
**

Supplement: Supplementary file 3 — Fig. S3. JUNB and CXCR4 protein exosomal expression comparison between Small Cell Lung Cancer (SCLC) patients' and Healthy Donors (HDs') plasma exosomes. [file MOL2-19-2038-s009.docx]
