# Supplementary material for: Integrative analysis of circulating tumor cells (CTCs) and exosomes from small‐cell lung cancer (SCLC) patients: a comprehensive approach
Source: Mol Oncol. 2024 Nov 22;19(7):2038–55. doi: 10.1002/1878-0261.13765 (PMC12234381; doi:10.1002/1878-0261.13765)
Supplement: Supplementary file 4 — Table S1. Number of Circulating Tumor Cells (CTCs) per phenotype and per patient, regarding CXCR4 and JUNB expression. [file MOL2-19-2038-s002.docx]

**Supplementary Table 1.** Number of Circulating Tumor Cells (CTCs) per phenotype and per patient, regarding CXCR4 and JUNB expression.

| SCLC patients’ CTCs | | | | | |
| --- | --- | --- | --- | --- | --- |
| Patient No. | **CK+CXCR4+JUNB+** | **CK+CXCR4-JUNB+** | **CK+CXCR4+JUNB-** | **CK+JUNB-CXCR4-** | **Total phenotypes per patient** |
| 1 | 0 | 1 | 1 | 3 | 3 |
| 2 | 2 | 17 | 0 | 18 | 3 |
| 3 | 1 | 22 | 1 | 4 | 4 |
| 4 | 4 | 2 | 1 | 23 | 4 |
| 5 | 0 | 3 | 0 | 1 | 2 |
| 6 | 5 | 0 | 1 | 3 | 3 |
| 7 | 2 | 2 | 0 | 20 | 3 |
| 8 | 1 | 0 | 2 | 2 | 3 |
| 9 | 5 | 0 | 0 | 6 | 2 |
| 10 | 2 | 0 | 0 | 0 | 1 |
| 11 | 0 | 0 | 2 | 105 | 2 |
| 12 | 4 | 6 | 2 | 31 | 4 |
| 13 | 0 | 1 | 2 | 2 | 3 |
| 14 | 2 | 0 | 1 | 2 | 3 |
| 15 | 1 | 2 | 12 | 33 | 4 |
| 16 | 2 | 3 | 1 | 60 | 4 |
| 17 | 0 | 1 | 0 | 0 | 1 |
| 18 | 1 | 0 | 1 | 6 | 2 |
| 19 | 0 | 0 | 0 | 0 | 0 |
| 20 | 0 | 0 | 0 | 0 | 0 |
| 21 | 2 | 6 | 3 | 33 | 4 |
| 22 | 0 | 0 | 0 | 0 | 0 |
| 23 | 0 | 0 | 1 | 1 | 2 |
| 24 | 0 | 1 | 0 | 1 | 2 |
| 25 | 0 | 2 | 0 | 0 | 1 |
| 26 | 0 | 0 | 0 | 0 | 0 |
| 27 | 0 | 0 | 0 | 0 | 0 |
| 28 | 0 | 0 | 0 | 0 | 0 |
| 29 | 0 | 2 | 0 | 1 | 2 |
| 30 | 3 | 6 | 1 | 5 | 4 |
| 31 | 11 | 5 | 14 | 6 | 4 |
| 32 | 1 | 0 | 1 | 0 | 2 |
| 33 | 2 | 1 | 2 | 1 | 4 |
| 34 | 4 | 7 | 6 | 10 | 4 |
| 35 | 9 | 5 | 2 | 2 | 4 |
| 36 | 2 | 5 | 1 | 3 | 4 |
| 37 | 4 | 5 | 1 | 0 | 3 |
| 38 | 2 | 1 | 3 | 2 | 4 |
| 39 | 2 | 3 | 2 | 1 | 4 |
| 40 | 0 | 6 | 0 | 6 | 2 |
| 41 | 0 | 2 | 0 | 3 | 2 |
| 42 | 0 | 1 | 0 | 0 | 1 |
| 43 | 0 | 1 | 0 | 2 | 2 |
| 44 | 0 | 1 | 0 | 0 | 1 |
| 45 | 0 | 0 | 0 | 0 | 0 |
| 46 | 2 | 2 | 0 | 6 | 3 |
| 47 | 0 | 0 | 0 | 0 | 0 |
| 48 | 1 | 0 | 0 | 0 | 1 |
| 49 | 0 | 1 | 0 | 2 | 2 |
| 50 | 0 | 0 | 0 | 2 | 1 |
| 51 | 0 | 0 | 2 | 1 | 2 |
| 52 | 0 | 0 | 0 | 0 | 0 |
| 53 | 0 | 0 | 0 | 0 | 0 |
| 54 | 0 | 0 | 1 | 1 | 2 |
| 55 | 0 | 0 | 0 | 0 | 0 |
| 56 | 0 | 0 | 0 | 0 | 0 |
| 57 | 0 | 0 | 2 | 2 | 2 |
| 58 | 0 | 0 | 0 | 1 | 1 |
| 59 | 1 | 0 | 1 | 1 | 3 |
| 60 | 0 | 0 | 0 | 0 | 0 |
| 61 | 1 | 0 | 1 | 0 | 2 |
| 62 | 0 | 0 | 0 | 2 | 1 |
| 63 | 0 | 0 | 0 | 0 | 0 |
| 64 | 0 | 0 | 0 | 1 | 1 |
| 65 | 1 | 0 | 0 | 1 | 2 |
| 66 | 0 | 0 | 1 | 1 | 2 |
| 67 | 0 | 1 | 0 | 3 | 2 |
| 68 | 0 | 0 | 0 | 0 | 0 |
| 69 | 0 | 0 | 0 | 0 | 0 |
| 70 | 1 | 0 | 0 | 0 | 1 |
| 71 | 0 | 0 | 0 | 0 | 0 |
| 72 | 0 | 0 | 0 | 0 | 0 |
| 73 | 0 | 0 | 0 | 0 | 0 |
| 74 | 0 | 0 | 0 | 0 | 0 |
| 75 | 1 | 0 | 0 | 0 | 1 |
| 76 | 0 | 0 | 2 | 1 | 2 |
| 77 | 1 | 0 | 0 | 0 | 1 |
| 78 | 0 | 1 | 0 | 2 | 2 |
| 79 | 0 | 0 | 0 | 0 | 0 |
| 80 | 0 | 0 | 0 | 0 | 0 |
| 81 | 1 | 2 | 1 | 4 | 4 |
| 82 | 2 | 2 | 3 | 3 | 4 |
| 83 | 0 | 0 | 0 | 0 | 0 |
| 84 | 0 | 0 | 1 | 1 | 2 |
| 85 | 0 | 0 | 0 | 0 | 0 |
| 86 | 4 | 1 | 0 | 4 | 3 |
| 87 | 1 | 1 | 0 | 0 | 2 |
| 88 | 1 | 2 | 0 | 0 | 2 |
| 89 | 1 | 0 | 0 | 0 | 1 |
| 90 | 0 | 0 | 0 | 0 | 0 |
| 91 | 0 | 0 | 0 | 0 | 0 |
| 92 | 0 | 0 | 1 | 0 | 1 |
| 93 | 0 | 1 | 2 | 1 | 3 |
| 94 | 1 | 0 | 0 | 0 | 1 |
| 95 | 0 | 0 | 0 | 0 | 0 |
| 96 | 3 | 3 | 1 | 1 | 4 |
| 97 | 0 | 0 | 1 | 0 | 1 |
| 98 | 0 | 0 | 0 | 0 | 0 |
| 99 | 0 | 0 | 0 | 0 | 0 |
| 100 | 0 | 0 | 0 | 0 | 0 |
