# Supplementary material for: Integrative analysis of circulating tumor cells (CTCs) and exosomes from small‐cell lung cancer (SCLC) patients: a comprehensive approach
Source: Mol Oncol. 2024 Nov 22;19(7):2038–55. doi: 10.1002/1878-0261.13765 (PMC12234381; doi:10.1002/1878-0261.13765)
Supplement: Supplementary file 5 — Table S2. Correlation between total Circulating Tumor Cells (CTCs) count per patient and the examined CTCs phenotypes regarding CXCR4 and JUNB. [file MOL2-19-2038-s001.docx]

**Supplementary Table 2.** Correlation between total Circulating Tumor Cells (CTCs) count per patient and the examined CTCs phenotypes regarding CXCR4 and JUNB.

| Total CTC number per patient | rho | *p* Value |
| --- | --- | --- |
| CK^+^CXCR4^+^JUNB^+^ | 0.688 | < 0.001 |
| CK^+^CXCR4^–^JUNB^+^ | 0.741 | < 0.001 |
| CK^+^CXCR4^+^JUNB^–^ | 0.660 | < 0.001 |
| CK^+^CXCR4^–^JUNB^–^ | 0.892 | < 0.001 |
