# Supplementary material for: Integrative analysis of circulating tumor cells (CTCs) and exosomes from small‐cell lung cancer (SCLC) patients: a comprehensive approach
Source: Mol Oncol. 2024 Nov 22;19(7):2038–55. doi: 10.1002/1878-0261.13765 (PMC12234381; doi:10.1002/1878-0261.13765)
Supplement: Supplementary file 6 — Table S3. Number of Circulating Tumor Cells (CTCs) per phenotype and per patient, regarding Programmed death‐ligand 1 (PD‐L1) expression. [file MOL2-19-2038-s005.docx]

**Supplementary Table 3.** Number of Circulating Tumor Cells (CTCs) per phenotype and per patient, regarding PD-L1 expression.

| **SCLC patients' CTCs** | | | |
| --- | --- | --- | --- |
| Patient No. | **CK^+^PD-L1^+^CD45^–^** | **CK^+^PD-L1^–^CD45^–^** | Total phenotypes per patient |
| **1** | 9 | 7 | 2 |
| **2** | 0 | 1 | 1 |
| **3** | 3 | 2 | 2 |
| **4** | 1 | 1 | 2 |
| **5** | 2 | 0 | 1 |
| **6** | 1 | 0 | 1 |
| **7** | 0 | 3 | 1 |
| **8** | 0 | 3 | 1 |
| **9** | 2 | 0 | 1 |
| **10** | 6 | 4 | 2 |
| **11** | 4 | 0 | 1 |
| **12** | 5 | 3 | 2 |
| **13** | 5 | 1 | 2 |
| **14** | 0 | 1 | 1 |
| **15** | 13 | 57 | 2 |
| **16** | 0 | 1 | 1 |
| **17** | 0 | 1 | 1 |
| **18** | 0 | 2 | 1 |
| **19** | 3 | 0 | 1 |
| **20** | 1 | 5 | 2 |
| **21** | 17 | 12 | 2 |
| **22** | 15 | 8 | 2 |
| **23** | 0 | 1 | 1 |
| **24** | 41 | 17 | 2 |
| **25** | 7 | 9 | 2 |
| **26** | 4 | 3 | 2 |
| **27** | 9 | 10 | 2 |
| **28** | 1 | 1 | 2 |
| **29** | 1 | 0 | 1 |
| **30** | 3 | 1 | 2 |
| **31** | 1 | 0 | 1 |
| **32** | 3 | 1 | 2 |
| **33** | 1 | 0 | 1 |
| **34** | 2 | 2 | 2 |
| **35** | 1 | 0 | 1 |
| **36** | 2 | 0 | 1 |
| **37** | 1 | 0 | 1 |
| **38** | 4 | 1 | 2 |
| **39** | 1 | 0 | 1 |
| **40** | 1 | 0 | 1 |
| **41** | 0 | 1 | 1 |
| **42** | 1 | 1 | 2 |
| **43** | 0 | 2 | 1 |
| **44** | 0 | 2 | 1 |
| **45** | 0 | 1 | 1 |
| **46** | 0 | 1 | 1 |
| **47** | 4 | 1 | 2 |
| **48** | 0 | 5 | 1 |
| **49** | 0 | 1 | 1 |
| **50** | 0 | 1 | 1 |
| **51** | 0 | 1 | 1 |
| **52** | 0 | 1 | 1 |
| **53** | 1 | 0 | 1 |
| **54** | 2 | 0 | 1 |
| **55** | 2 | 1 | 2 |
| **56** | 1 | 0 | 1 |
| **57** | 0 | 1 | 1 |
