# Supplementary material for: Integrative analysis of circulating tumor cells (CTCs) and exosomes from small‐cell lung cancer (SCLC) patients: a comprehensive approach
Source: Mol Oncol. 2024 Nov 22;19(7):2038–55. doi: 10.1002/1878-0261.13765 (PMC12234381; doi:10.1002/1878-0261.13765)
Supplement: Supplementary file 7 — Table S4. Correlation between total Circulating Tumor Cells (CTCs) count per patient and the examined CTC phenotypes regarding Programmed death‐ligand 1 (PD‐L1) expression. [file MOL2-19-2038-s008.docx]

**Supplementary Table 4.** Correlation between total Circulating Tumor Cells (CTCs) count per patient and the examined CTC phenotypes regarding PD-L1.

| Total CTC number per patient | rho | *p* Value |
| --- | --- | --- |
| CK^+^PD-L1^+^CD45^–^) | 0.814 | < 0.001 |
| CK^+^PD-L1^–^CD45^–^ | 0.817 | < 0.001 |
